# Genetic Background of Kirgiz Ethnic Group From Northwest China Revealed by Mitochondrial DNA Control Region Sequences on Massively Parallel Sequencing

**DOI:** 10.3389/fgene.2022.729514

**Published:** 2022-02-23

**Authors:** Hongdan Wang, Man Chen, Chong Chen, Yating Fang, Wei Cui, Fanzhang Lei, Bofeng Zhu

**Affiliations:** ^1^ Key Laboratory of Shaanxi Province for Craniofacial Precision Medicine Research, College of Stomatology, Xi’an Jiaotong University, Xi’an, China; ^2^ Medical Genetic Institute of Henan Province, Henan Provincial People’s Hospital, Zhengzhou University People’s Hospital, Zhengzhou, China; ^3^ Guangzhou Key Laboratory of Forensic Multi-Omics for Precision Identification, School of Forensic Medicine, Southern Medical University, Guangzhou, China; ^4^ Multi-Omics Innovative Research Center of Forensic Identification, Department of Forensic Genetics, School of Forensic Medicine, Southern Medical University, Guangzhou, China; ^5^ College of Forensic Medicine, Xi’an Jiaotong University, Xi’an, China

**Keywords:** mitochondrial DNA control region, massively parallel sequencing, Kirgiz ethnic group, genetic polymorphism, population evolution

## Abstract

The mitochondrial DNA (mtDNA) has been used to trace population evolution and apply to forensic identification due to the characteristics including lack of recombination, higher copy number and matrilineal inheritance comparing with nuclear genome DNA. In this study, mtDNA control region sequences of 91 Kirgiz individuals from the Northwest region of China were sequenced to identify genetic polymorphisms and gain insight into the genetic background of the Kirgiz ethnic group. MtDNA control region sequences of Kirgiz individuals presented relatively high genetic polymorphisms. The 1,122 bp sequences of mtDNA control region could differ among unrelated Kirgiz individuals, which suggested the mtDNA control region sequences have a good maternal pedigree tracing capability among different Kirgiz individuals. The neutrality test, mismatch distribution, Bayesian phylogenetic inference, Bayesian skyline analysis, and the median network analyses showed that the Kirgiz group might occurred population expansion, and the expansion could be observed at about ∼53.41 kilo years ago (kya) when ancestries of modern humans began to thrive in Eurasia. The pairwise population comparisons, principal component analyses, and median network analyses were performed based on haplogroup frequencies or mtDNA control region sequences of 5,886 individuals from the Kirgiz group and the 48 reference populations all over the world. And the most homologous haplotypes were found between Kirgiz individuals and the East Asian individuals, which indicated that the Kirgiz group might have gene exchanges with the East Asian populations.

## Introduction

The Kirgiz group is one of the official minority ethnic group in China. According to the seventh census, there are about 186 thousand Kirgiz individuals in China, which are mainly located in the Northwest China. Additionally, hundreds of Kirgiz individuals are settled in Chinese Heilongjiang Province. The Kirgizs speak Kepchak, which is a subgroup of the Turkic group of Altaic language family. In China, the history of the Kirgiz group can be traced back to the period of Emperor Wu of the Western Han Dynasty (109–91 BC), and since then the Kirgizs have the appearance characteristics of both European and East Asian people according to the historical record (Gordon, 2009). Later, Kirgiz ancestors gradually expanded geographically and spatially, and experienced the “Kigu”, “Pikasi”, and “Bulgari” periods successively during Tang Dynasty and Qing Dynasty (Abramzon and Tabyshaliev, 1990).

In previous studies researchers Guo et al. (Guo et al., 2018), Xie et al. (Xie et al., 2020) and Zhang et al. (Zhang et al., 2021) clarified the genetic background of Kirgiz group based on insertion/deletion (InDel), including the 30 commercial InDel system and self-developed 39 ancestry informative marker (AIM) InDel system, respectively. The above mentioned researches demonstrated that Kirgiz group had the relatively close genetic distances with Kazakh and Hui groups based on autosomal InDel genetic markers. Wang et al. (2019) explored the genetic diversity of Kirgiz group based on the presence/absence polymorphisms of killer cell immunoglobulin like receptor genes. And the research indicated that the Kirgiz group represented small genetic differences with populations speaking the same family language. The genetic population studies were conducted by Guo et al., Song et al., and Chen et al., they studied the genetic diversity distributions of Kirgiz group on basis of 60 single nucleotide polymorphisms (SNPs) in mtDNA and 24 Y chromosomal short tandem repeats (Y-STRs) (Guo et al., 2020); 17 Y chromosomal SNPs (Y-SNPs) and 27 Y-STRs (Song et al., 2021); and 23 autosomal-STRs (Pengyu Chen et al., 2019), respectively. And they concluded that the Kirgiz group had the genetic admixture of East Asia and Europe after comparing with the other continental populations.

The control region, also known as the D-loop region, is a sequence of 1,122 bp on the mtDNA, including two segments at 1-576 and 16024-16569, respectively (Anderson et al., 1981). The multiple copies of mtDNA, and the resistance to degradation with the circular structure make mtDNA more suitable for forensic trace and degraded samples than nuclear DNA (Gallimore et al., 2018; Amorim et al., 2019). The high mutation rates of mtDNA sequences make that the sequences are high polymorphisms especially in the control region. And the highly polymorphic mtDNA control region sequences have the potential to distinguish unrelated individuals (Strobl et al., 2019; Ta et al., 2021). The characteristics of the mtDNA maternal inheritance can be used to track the maternal family (Bandelt and Dür, 2007; Parson et al., 2014), and can also be used to track the genetic relationships among the Kirgiz group and other reference populations.

Based on the massively parallel sequencing (MPS) platform, the influences of multiple copies of mtDNA and the homologous fragments of nuclear DNA make that the mtDNA data often require deeper sequencing depth, and well-balanced sequencing read and amplicon to ensure the obtained reliable results (Amorim et al., 2019). In addition, having specific mtDNA control region sequences among different maternal pedigrees is the prerequisite basis for forensic maternal tracing application. And the above-mentioned characteristics would be reflected in the high genetic diversities among unrelated individuals (Scally, 2016). This study was carried out to evaluate the forensic application efficiencies of the mtDNA control region sequences in the Kirgiz group, and to uncover possible historical events in the Kirgiz origin, and to disclose the genetic affinities between Kirgiz and Chinese other populations or international populations. In this study, the mtDNA control region sequences in 91 unrelated healthy individuals from Kirgiz group were sequenced to analyze the genetic structure of Kirgiz group based on the maternal genetic materials. Furthermore, the mtDNA control region sequences of 5,795 individuals from 48 previously published populations were also collected to compare genetic differences among populations and gain insight into the genetic background of Kirgiz group.

## Materials and Methods

### Sample Collection

**FIGURE 1:**
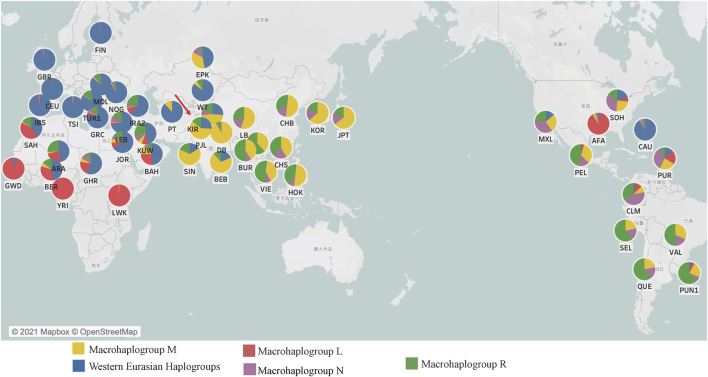
The macrohaplogroup distributions and the geographical coordinates of the Kirgiz group and 48 reference populations around the world. In each pie chart, the red showed the Africa specific macrohaplogroup L, the yellow showed the commonly observed macrohaplogroup M in East Asian populations, the blue indicated the Western Eurasian haplogroup, the purple showed the macrohaplogroup N and the green showed the macrohaplogroup R. Within the macrohaplogroups N and R of this figure, the Western Eurasian specific haplogroups had been excluded.

In total, bloodstains within FTA™ cards were collected from 91 unrelated healthy individuals of the Kirgiz group in the Northwest region of China. The detailed sampling locations of all 49 populations including Kirgiz group and 48 reference populations were shown on the world map in Figure 1. All participants were informed in details of the content and purpose of the present study, and all signed the written informed consents. This study was approved by the Ethics Committee of Xi’an Jiaotong University Health Science Center (Approval Number: XJTULAC201) and complied with the ethical principle of the World Medical Association Declaration of Helsinki (World Medical Association, 2013). According to the questionnaire survey, the selected donors of random sampling were all local residents and their immediate family members (parents, grandparents, and maternal grandparents) were all Kirgiz individuals. To protect the privacy of volunteers, all samples were numbered and anonymized during the whole experiments.

### Library Preparation and Sequencing

Genome mtDNA of the 91 bloodstain samples was extracted with the PrepFiler BTA™ forensic DNA extraction kit (Thermo Fisher Scientific, MA, United States). The magnetic bead method was used for DNA extraction by the above kit. The extraction method selected in this study combined uniquely structured magnetic particles and the optimized multi-component chemistry surface, to provide efficient DNA binding capacity and high DNA recovery rate. The extracted genome DNA was quantified with the Qubit™ dsDNA HS assay kit (Thermo Fisher Scientific) at the Qubit™ four Fluorometer (Thermo Fisher Scientific). The mtDNA control region sequences of 91 unrelated individuals and positive control samples (007 and 9947A) were amplified using the Precision ID mtDNA Control Region Panel (Thermo Fisher Scientific). The libraries were prepared with Precision ID DL8 Kit (Thermo Fisher Scientific) on Ion Chef™ System (Thermo Fisher Scientific). The mtDNA control region was amplificated with 14 primer pairs in two primer pools. The libraries were quantified using the Ion Library TaqMan™ Quantitation Kit (Thermo Fisher Scientific) on the 7,500 Real-Time PCR System (Thermo Fisher Scientific). The sequencing templates were prepared using with the Ion S5™ Precision ID Chef & Sequencing Kit (Thermo Fisher Scientific), and were loaded to the Ion 530™ Chip (Thermo Fisher Scientific) on the Ion Chef™ System (Thermo Fisher Scientific). The sequencing runs were accomplished using the Ion S5™ Precision ID Chef & Sequencing Kit (Thermo Fisher Scientific) on Ion S5™ XL System (Thermo Fisher Scientific). All the above experimental procedures were performed strictly according to the manufacturer’s recommendations.

### Data Analyses

The raw sequencing data were analyzed using Converge ™ Software V2.2 (Thermo Fisher Scientific) of MPS mtDNA module with HID Genotyper 2.2 of default parameters. After aligning to the Revised Cambridge Reference Sequence (rCRS) (Andrews et al., 1999), all the variations were called for further scoring. After the mutations were verified, they were mapped to nodes in the Phylotree 17 (http://www.phylotree.org/tree/index.htm) of human mitochondrial DNA lineages, further allocated the haplotypes to the closest haplogroups, which were submitted to the EMPOP database for quality control (Parson and Dür, 2007). The haplotypes were annotated according to the recommendations of International Society of Forensic Genetics with the nomenclature rules of mtDNA typing (Parson et al., 2014). The analytical threshold at all 1,122 sequences was 20×, and the thresholds of confirmed mutation, point heteroplasmy, insertion and deletion (length heteroplasmy) were 96%, 10%, 20%, and 30% of the total reads with each variant, respectively.

### Statistical Analyses

A total of 5,795 individuals from 48 reference populations distributed all over the world were chosen for further investigation of the genetic relationships among the studied Kirgiz ethnic group and the reference populations based on the mtDNA control region sequences. The sample size of each selected population was more than 30 individuals. The 48 reference populations included seven populations from Africa, five populations from North America, six populations from South America, nine populations from Europe, four populations from Central Asia, eight populations from East Asia, four populations from South Asia, two populations from Southeast Asia and four populations from West Asia. The detailed information of the Kirgiz group and these 48 reference populations was showed in Supplementary Table S1.

To evaluate the sequencing performance, we calculated several parameters including the true allelic ratio, noise ratio, ratio of two sequencing directions and the mean depth of each sample, the average depth of each amplicon, and the depth ratio between two primer pools of each sample. The true allele ratios were calculated by dividing the read depth of the true allele into total read depth of each sample, and the remaining undefined reads were defined as noise. The sequencing ratios were calculated by dividing the coverage of 5′ reads into the 3’ reads. The mean depth per sample and average depth of each amplicon were also calculated directly. The depth ratios were by dividing the lowest read depth into the greatest read depth of each sample.

The haplogroup frequencies based on the mtDNA control region sequences of 91 Kirgizs were calculated directly. The phylogenetic tree for 91 Kirgiz mtDNA control region sequences was performed using the online tool of HaploGrep 2 v2.0 (https://haplogrep.i-med.ac.at/app/index.html) under the Kulczynski measure. The example case of U1a2 with detailed calculation formula could be found at the website https://haplogrep.i-med.ac.at/2018/06/21/explaining-the-formula/. These mutations including 309.1C(C), 315.1C, 515-522 InDel of AC, A16182C, A16183C, 16193.1C(C), C16519T and T16519C were excluded when the phylogenetic tree was reconstructed, and the tree was showed in Supplementary Figure S1.

DNA Sequence Polymorphism v6 (DnaSP v6) (Librado and Rozas, 2009) was used to calculate the statistical parameters of the 91 mtDNA control region sequences, which included the number of polymorphic site (S), haplotype diversity (Hd), nucleotide diversity (Pi), and the average number of nucleotide difference (k). Analysis of molecular variation (AMOVA), neutrality tests (Tajima’s D and Fu’s Fs tests), and mismatch distribution (the sum of square deviation, SSD, and Harpending’s raggedness index, R) were performed with mtDNA control region sequences using Arlequin 3.5.1.3, simultaneously (Excoffier and Lischer, 2010). The Bayesian phylogenetic inference and Bayesian Skyline Plot (BSP) of Kirgiz group were performed using BEAST 2.6.5 software to deduce the time when the Kirgiz group expansion occurred (Bouckaert et al., 2019). The nucleotide substitution model of TN93 and gamma site model with the substitution rate of 1.57E-8 of the single nucleotide substitution per year were set (Soares et al., 2009), and the strict clock model was selected. The pairwise divergence time for *Homo sapiens* and *Homo* neanderthalensis was chosen to calibrate the divergence time, as a median time of 0.55 (±0.054) million years ago (MYA) (Soares et al., 2009). The adaptive Monte Carlo Markov chain (MCMC) approach was used to evaluate the molecular evolution of the mtDNA control region sequences, the MCMC chain length was 1,000,000, and auto optimize displayed for 10,000 steps. Bayesian Skyline Plot (BSP) reconstruction was performed using Tracer 1.7.0 (Rambaut et al., 2018) and the phylogenetic tree was annotated by FigTree v1.4.4 (http://tree.bio.ed.ac.uk/software/figtree/). The detailed geographical coordinates and the macrohaplogroup compositions of all the populations were visualized using Tableau Desktop v 2021.1. Heatmap was constructed using the ‘heatmap’ package of *R* statistical software based on the pairwise *F*_*ST*_ values between the Kirgiz group and 48 reference populations (https://www.r-project.org/). A neighbor-joining (NJ) tree was built using Molecular Evolutionary Genetics Analysis (Mega) v10.2.1 based on the pairwise *F*_*ST*_ values among 49 populations. And the NJ tree was displayed and annotated using the online tool iTOL v 6.1.2 (https://itol.embl.de/itol.cgi). The two-dimensional (2D) and three-dimensional (3D) principle component analyses (PCA) were constructed using the dimensionality reduction analysis module of SPSS Statistics v.19.0 based on the haplogroup frequencies generated from mtDNA control regions of the Kirgiz group and 48 reference populations. The haplogroup network calculations were performed using Network v.10.2.0.0 of the median joining module based on the mtDNA control region sequences from the Kirgiz and the 48 reference populations, finally, of which 1,403 individuals from the 48 reference populations sharing the same or similar haplogroups with the target Kirgiz individuals were selected to participate in the network relationship evaluations. The following network plots were visualized with the network publisher (https://www.fluxus-engineering.com/nwpub.htm).

## Results

### General Performance of the Sequencing Results

**FIGURE 2:**
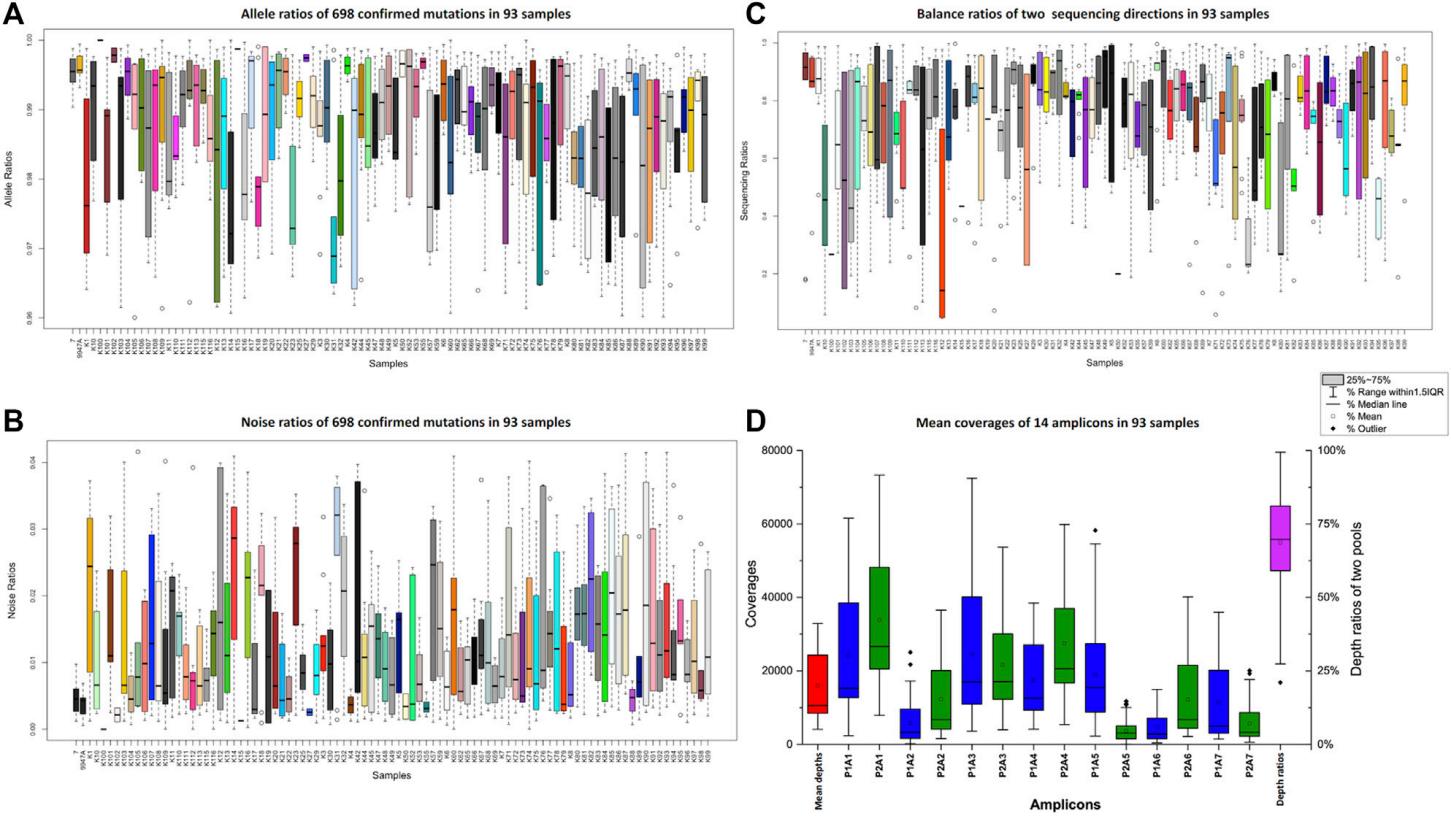
Ratios of true alleles **(A)**, ratios of noises **(B)**, balance ratios of two different sequencing directions **(C)** and the average depths of 14 amplicons **(D)** of the 91 tested Kirgiz individuals and two positive control samples. The 007 and 9947A were showed at the first two boxplots within Figure 2. The series of blue and green boxplots in Figure 2 represented the 14 amplicons, the red box plot showed the average depths of the whole panel in 91 Kirgiz individuals, and the purple one showed the balance ratios of two pools in 91 Kirgiz individuals and two positive control samples.

All the mtDNA control region sequences from the 91 Kirgiz individuals and the positive control samples (9947A and 007) were successfully generated from four sequencing runs on the Ion S5™ XL system. The true allelic ratios and noise ratios were shown in Figures 2A,B. Even when outliers were taken into consideration, the true alleles of all tested samples covered more than 96% of sequencing reads, while the noise was less than 4%. The balance ratios of all the amplicons from two directions in the 91 samples were shown in Figure 2C. The balance parameters of most samples were around 0.5, which indicated the relatively good performance of the bi-directional sequencing. In Figure 2D, the blue and green bars represented the average depths of two primer pools with 14 amplicons when the control region of mtDNA was amplified using the imbrication strategy. The mean depth per individual (the first red boxplot in Figure 2D) was 12989 ± 10865 × (mean ± standard deviation). The mean depths of the first pool ranged from 4,598 × (P1A6) to 24535 × (P1A3) which were shown in the blue boxplot in Figure 2D. The mean depths of the second pool ranged from 3,632 × (P2A5) to 33856 × (P2A1), as shown in the green boxplot in Figure 2D. The average depth ratio of two pools (the purple boxplot in Figure 2D) was 68.65%.

### Analyses at the Intra-Population Level

#### Genetic Variants of mtDNA Control Region Sequences Observed in Kirgiz Group

The phylogenetic tree of mtDNA control region mutations generated from 91 unrelated Kirgiz individuals was performed using the online tool HaploGrep v.2.0 based on the latest mtDNA tree Build 17, and the result was shown in Supplementary Figure S1. All samples could be distinguished between each other based on mtDNA control region sequence variations. In total, 168 variants were observed among 91 unrelated Kirgiz individuals including 52 local and three unexpected global mutations (199M, 451G, 502 del), the global mutations had never been reported previously in the EMPOP database. The details of the 168 mutations and the mutation rates were shown in Supplementary Table S2. The variants including 263G, 73G, 16223T, 489C and 16189C were the first five most common mutation points identified in Kirgiz group when compared with the rCRS.

### Haplogroup Allocations and Forensic Parameters of mtDNA Control Region Sequences in Kirgiz Group

**FIGURE 3:**
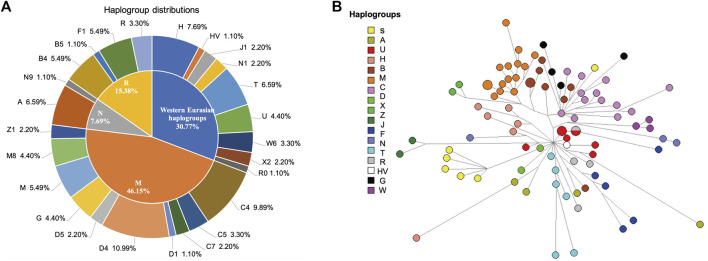
The haplogroup distribution **(A)** and median network **(B)** of the mtDNA control region sequences of 91 Kirgiz individuals. In **(A)**, the macrohaplogroups R, M and N were commonly observed in East Asia, and the Western Eurasian haplogroups were commonly observed in Europe. In **(B)**, the median network analysis among Kirgiz individuals was conducted using the mtDNA control region sequences originated from 91 Kirgiz individuals. Different colors represented different haplogroups and the same haplogroups clustered together and separated with others.

A total of 66 haplogroups and 81 haplotypes were identified from 91 Kirgiz unrelated individuals based on mtDNA control region sequences. The variants (including single point mutation, InDel, and heteroplasmy were marked in green, grey and yellow, respectively), haplotypes and allocated haplogroups of 91 Kirgiz individuals were shown in Supplementary Table S3. The mtDNA haplogroup distributions of the Kirgiz group were shown in Figure 3A. The haplogroups of 91 Kirgizs comprised of 69.23% East Eurasian haplogroups and 30.77% Western Eurasian haplogroups. The macrohaplogroup M (2.20% of Z1, 4.40% of M8, 5.49% of M, 4.40% of G, 2.20% of D5, 10.99 of D4, 1.10% of D1, 2.20% of C7, 3.30% of C5, and 9.89% of C4), macrohaplogroup R (5.49% of F1, 1.10% of B5, 5.49% of B4, and 3.30% R) and macrohaplogroup N (1.10% of N9, 6.59% of A) commonly observed in East Eurasia populations were detected in the Kirgiz group, respectively. Additionally, the Western Eurasian haplogroups including H (7.69%), HV (1.10%), J1 (2.20%), N1 (2.20%), N1 (2.20%), T (6.59%), U (4.40%), W6 (3.30%), X2 (2.20%), and R0 (1.10%) were also observed in the Kirgiz group.

DNA polymorphisms of the studied Kirgiz group were evaluated, and the results of indexes were presented as follow, number of polymorphic site (S: 107), number of haplotype (h: 81), haplotype diversity (Hd: 0.997), nucleotide diversity (Pi: 0.00842) and the average number of nucleotide difference (k: 9.017), these parameters indicated the relatively high genetic diversities of mtDNA control region sequences was in Kirgiz group. The Tajima’s D test (-1.950, *p*-value < 0.05) and Fu’s FS test (-25.247, *p*-value < 0.05) of mtDNA variants for the 91 Kirgiz individuals were both calculated to be relatively large negative values, and the results might indicate that the variants were significantly deviated from neutral mutations.

### Population Expansion of the Kirgiz Group

**FIGURE 4:**
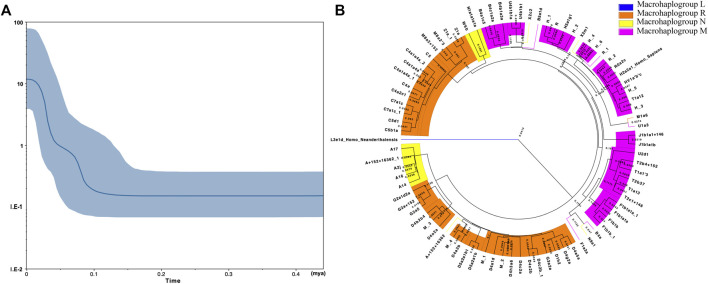
The BSP plot **(A)** and the Bayesian phylogenetic tree **(B)** generated from the mtDNA control region sequences of 91 Kirgiz individuals and two reference sequences (*Homo sapiens* and *Homo neanderthalensis*). In **(A)**, the X - axis represented the expansion time (million years ago for each unit) while the Y - axis showed the assumed effective population size on a logscale. The blue line in the bold represented the median population size. The blue shade showed the boundary of the 95% highest posterior density. The phylogenetic tree shown in **(B)** was reconstructed on basis of the tree file which was generated from the process of expansion time evaluation with BEAST 2.6.5 by the FigTree v1.4.4.

The mismatch distribution graph shown in Supplementary Figure S2 revealed a single peak, which indicated the Kirgiz group might occur the population expansion event according to the previous research method (Slatkin and Hudson, 1991). The SSD and Raggedness indexes of two models in the Kirgiz group were 0.00182 (SSD, *p*-value = 0.60000), 0.00689 (Raggedness index, *p*-value = 0.73000) for the sudden expansion model, 0.00216 (SSD, *p*-value = 0.57000) and 0.00689 (Raggedness index, *p*-value = 0.86000) for the spatial expansion model, respectively. The median network calculation was performed based on the mtDNA control region sequences of the 91 Kirgiz individuals, and the network plot of 91 Kirgiz individuals was shown in Figure 3B. Small branches belonged to the same haplogroup clustered together and separated from the other different haplogroup branches. Furthermore, as shown in Figure 4, Bayesian Skyline Plot (BSP) analysis was conducted based on the ancestral theory to quantify the evolutionary background of population size and history**.** The expansion time with the largest slope of the BSP abscissa was directly read. And the BSP abscissa showed that the Kirgiz group had grown at about *53.41 kya* (Figure 4A, the abscissa corresponding to the dash line). The annotated phylogenetic tree (Figure 4B) showed the estimated divergence time between *Homo sapiens* and *Homo* neanderthalensis was 0.5413 MYG, which was very close to the expected time of 0.55 MYG.

### Genetic Relationship Analyses Among Kirgiz Group and 48 Reference Populations

As for an important genetic marker for population evolution analysis and ancestral information inference, mtDNA control region sequences originated from 91 Kirgiz individuals were combined with other 5,795 mtDNA control region sequences from those individuals of 48 reference populations, which were used to analyze genetic relationships between Kirgiz group and these reference populations. The detailed geographical coordinates and the macrohaplogroup compositions of the Kirgiz group and 48 reference populations were shown in Figure 1 and Supplementary Table S4. As shown in Figure 1, with the advancement towards Africa, Europe, Asia, and America, the proportion of African-specific macrohaplogroup L gradually decreased and disappeared, and the proportions of macrohaplogroups M, N and R gradually increased. In macrohaplogroups N and R in this figure, the Western Eurasian specific haplogroups had been excluded. The 92.77% haplogroups of Caucasian population from North America (CAU) belonged to the Western Eurasian haplogroup because of the European origin.

### Genetic Distance Analyses Based on the Haplogroup Frequencies of mtDNA Control Region Sequences

**FIGURE 5:**
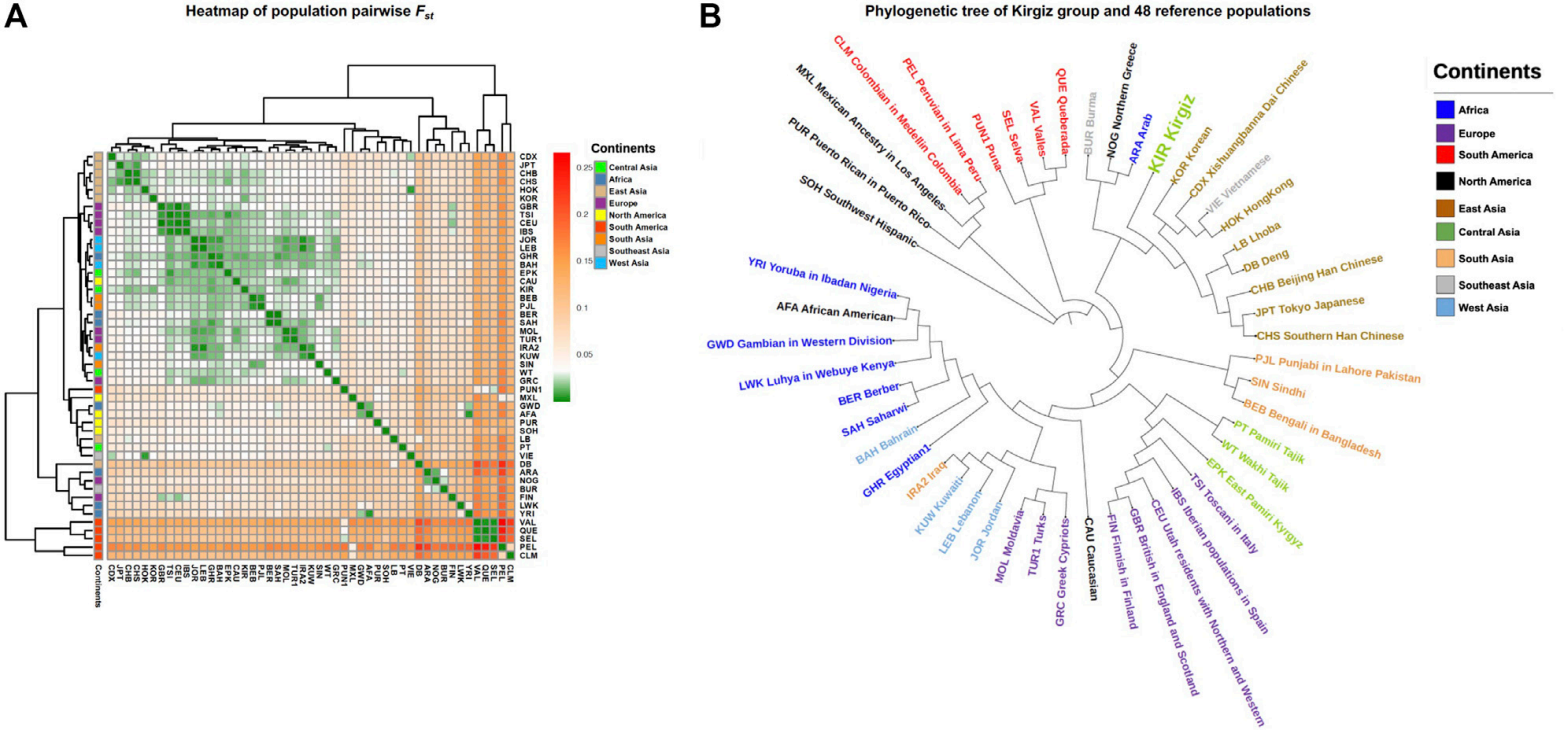
The *F_ST_* genetic distances and genetic relationships between Kirgiz group and other 48 reference populations. All the populations of different continents were labelled by different colors. **(A)** represented the heatmap of pairwise F_ST_ values between Kirgiz group and the 48 reference populations. *F_ST_* colors were from green, white, orange to red in turn, the corresponding values changed from small to large. **(B)** showed the phylogenetic tree of Kirgiz group and the reference populations based on pairwise *F_ST_* values.

Pairwise *F*_*ST*_ values were calculated based on the haplogroup frequencies of mtDNA control region sequences to obtain insight into the genetic affinities of the Kirgiz group and 48 reference populations. A population clustering heatmap of the pairwise *F*_*ST*_ values was plotted and shown in Figure 5A. In addition, the NJ tree of these 49 populations was displayed in Figure 5B. In the NJ tree, the Kirgiz group gathered together with other East Asia populations and formed a larger cluster. As a whole, the NJ tree indicated that most geographically adjacent populations gathered together. However, some populations were outliers which might be due to the population histories or the deviations caused by the different methodologies. The pairwise *F*_*ST*_ values were shown in Supplementary Table S6. The pairwise *F*_*ST*_ values between the Kirgiz group and other reference populations ranged from 0.01142 to 0.1499, and the median was 0.02836. The largest three *F*_*ST*_ values with the Kirgiz group were identified in three populations including Peruvian in *Lima* (PEL), Valles (VAL) and Colombian in Medellin (CLM). Among Chinese nine populations, the smallest three *F*_*ST*_ values were observed between Kirgiz and Beijing Han Chinese (CHB), East Pamiri Kyrgyz (EPK) and Southern Han Chinese (CHS).

### Principal Component Analyses Based on Haplogroup Frequencies

**FIGURE 6:**
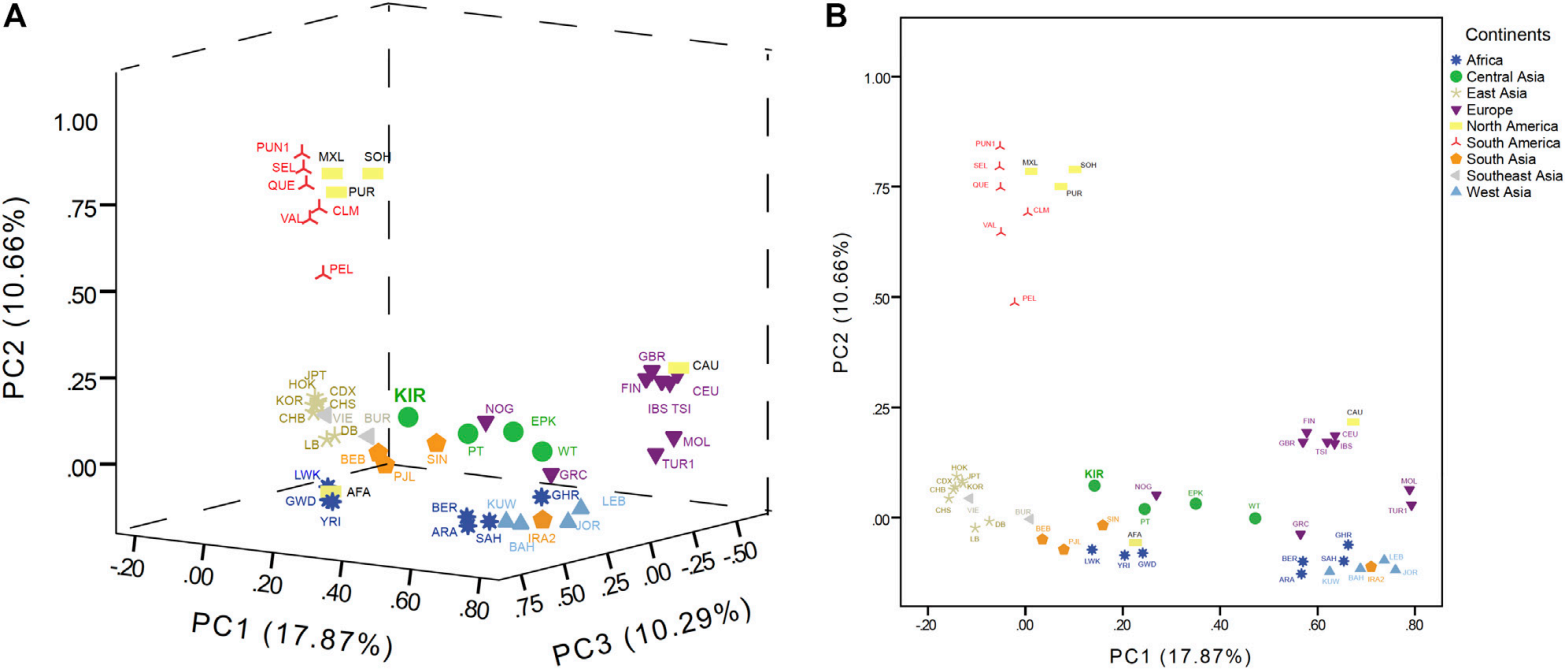
The 3D (PC1, PC2, and PC3) in **(A)** and 2D (PC1 and PC2) in **(B)** of PCA plots among the Kirgiz and the 48 reference populations.

PCA was conducted to evaluate the genetic affinities among Kirgiz group and reference populations based on the haplogroup frequencies in terms of maternal inheritance, and the haplogroup distributions of Kirgiz group and 48 reference populations were shown in Supplementary Table S5. And the scree PCA plot was shown in Supplementary Figure S3, which represented the principal components and percentages of explained variances. The first three principal components could explain 38.82% of the total variation among these 49 populations, while the first principal component explained 17.87% variation, the second explained 10.66%, and the third explained 10.29%. Based on the first three principal components, a 3D-PCA (PC1, PC2 and PC3) and a 2D-PCA (PC1 and PC2) were constructed and presented in Figure 6. The populations from nine different continental regions were marked with nine different colors. The PCA plots in Supplementary Figure S4 showed the 2D-PCA plots (PC1 and PC3, PC2 and PC3). As displayed in the PCA plots, the East Asia populations could be separated in PC1, the American populations could be separated in PC2, and the African populations could be separated in PC3.

### Haplogroup Median Network Analyses of the Observed Haplogroups in Kirgiz Group

**FIGURE 7:**
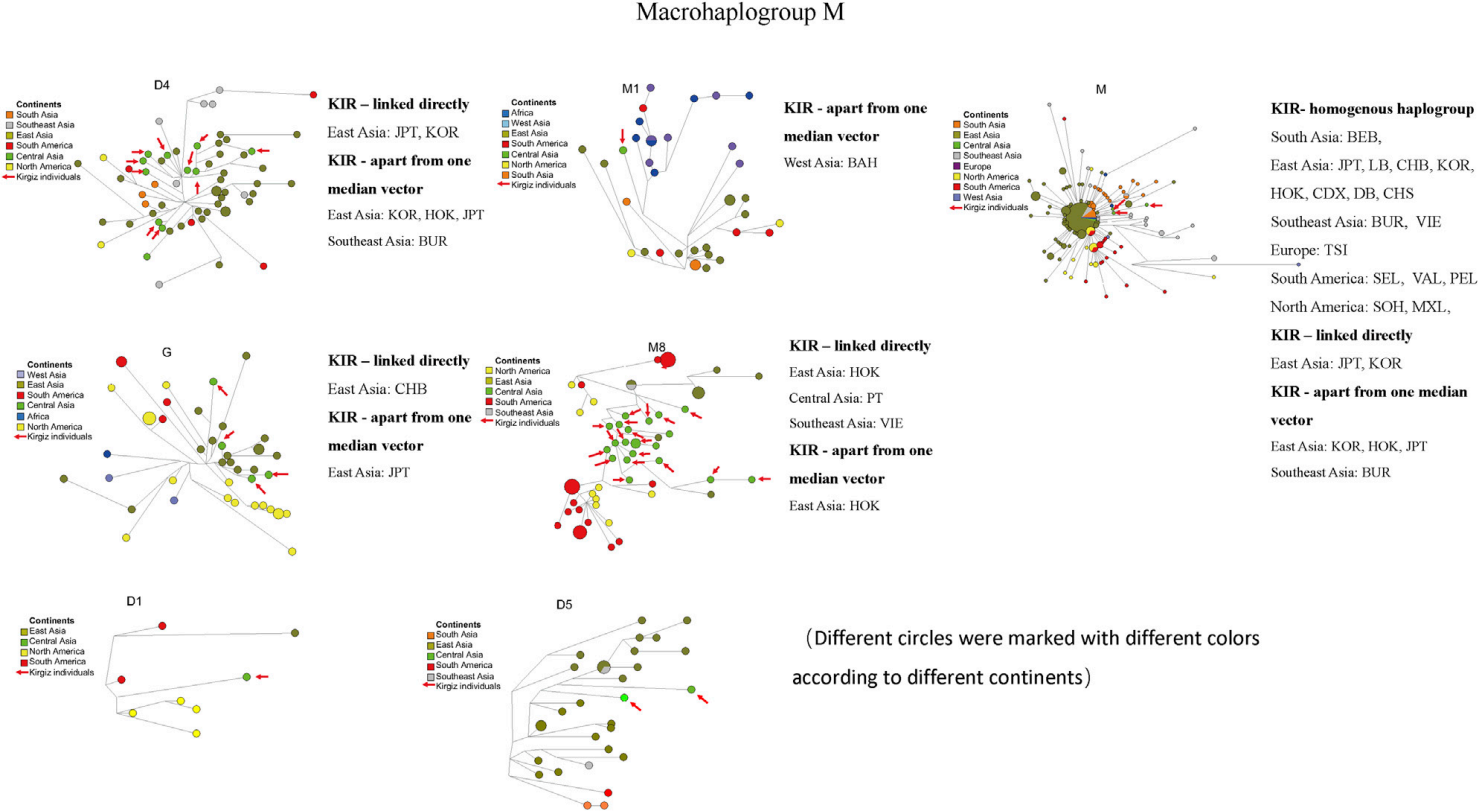
Median network of macrohaplogroup M. Different continents were labelled by different colors. There were 460 individual sequences of macrohaplogroup M which were allocated into seven haplogroups (D4, M1, M, G, M8, D1 and D5). The red arrows represented the haplotype sequences of the 42 Kirgiz individuals. KIR was the abbreviation of Kirgiz group.

To evaluate the network relationships between Kirgiz and other reference populations, all the 91 Kirgiz mtDNA control region sequences were compared with the individual sequences from 48 reference populations, and six median network graphs were finally generated. In total, 460 different individual sequences of macrohaplogroup M were distributed into seven different haplogroups, as 62 of D4, 36 of M1, 216 of M, 44 of G, 63 of M8, eight of D1, and 31 of D5, respectively. The median network diagrams of macrohaplogroup M with the largest proportion of Kirgiz group were labeled by different colors for different geographical locations, and different haplogroups were shown in Figure 7 and Supplementary Figure S5, respectively. In Figure 7, the red arrows indicated the 42 Kirgiz individual sequences were allocated into macrohaplogroup M. There were 58 individuals from eight populations from East Asia, one population from South Asia, two populations from Southeast Asia, one population from Europe, three populations from South America and two populations from North America which had homogenous haplogroup M with three Kirgiz individuals. Two East Asia populations were observed the direct link with Kirgiz group. Haplotype sequences apart from one median vector were observed between the Kirgiz individuals and one Southeast Asian, three East Asian individuals.

**FIGURE 8:**
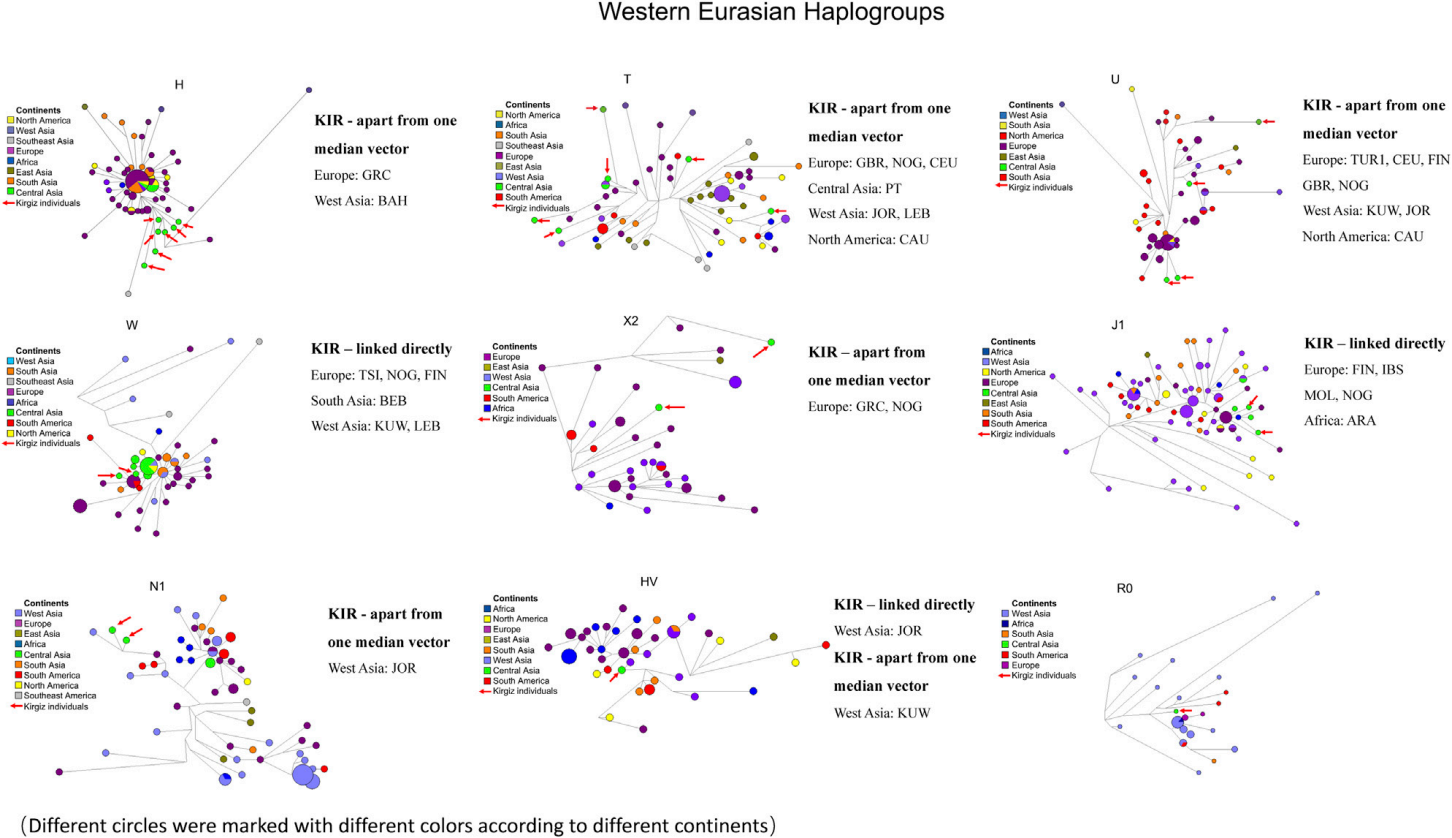
Median network of Western Eurasian haplogroup. Different continents were labelled by different colors. There were 582 individual sequences of Western Eurasian haplogroups which were allocated into nine haplogroups (H, T, U, W, X2, J1, N1, HV and R0). The red arrows represented the haplotype sequences of 28 Kirgiz individuals. KIR was the abbreviation of Kirgiz group.

The commonly observed Western Eurasian haplogroup including the H, T, U, W, X2, J1, N1, HV and R0 was the second-largest haplogroup in the Kirgiz group. These 27 Kirgiz individuals were allocated into this category. The Western Eurasian haplogroup of 27 Kirgiz individuals was allocated into nine above-mentioned haplogroups and nine different network plots labeled with colors for different continents (Figure 8) and different haplogroups (Supplementary Figure S6), respectively. There were 582 mtDNA control region sequences including in these nine median network relationships: 80 of H, 77 of T, 65 of U, 64 of W, 42 of X2, 97 of J1, 74 of N1, 47 of HV, and 36 of R0. Compared with the Kirgiz group, the directly linked individuals were observed in five European populations including Toscani in Italy (TSI), Northern Greece (NOG), Finnish in Finland (FIN), Iberian populations in Spain (IBS) and Moldavia (MOL), two West Asia populations i.e. Kuwaiti (KUW) and Jordan (JOR), one South Asia group Bengali in Bangladesh (BEB), one South America population Lebanon (LEB) and one African population Arab (ARA). Individuals apart from one median vector with Kirgizs were mostly observed in European populations, followed by the Central Asia and West Asia populations.

The 14 individuals of Kirgiz group were allocated into the macrohaplogroup R. Combining with 263 individuals from reference populations, 277 mtDNA control region sequences were distributed into four different haplogroups. And these four different network plots were shown in Supplementary Figure S7. These four different networks were marked by different colors for different continents in Supplementary Figure S7A, whereas, the different haplogroups were labeled by different colors in Supplementary Figure S7B, respectively. The median networks of four haplogroups B4, B5, R, and F1 were reconstructed based on the 91, 82, 70, and 20 mtDNA control region sequences among 48 populations, respectively. One individual of Utah resident with Northern and Western European ancestry (CEU) shared the homogenous haplogroup with a Kirgiz individual. Some individuals from eight populations including seven populations of Asia and one of Europe linked directly with 14 Kirgiz individuals, respectively. Seven individuals from three South Asian populations and one Southeast Asian, one North American, one South American, and one African population were found to be apart from one median vector with 14 Kirgiz individuals, respectively.

The smallest macrohaplogroup proportion of 91 unrelated Kirgiz individuals was N macrohaplogroup. The 25 sequences from the reference populations and seven sequences from the Kirgiz group were used to draw the median network diagram of the N macrohaplogroup. And the diagram of each haplogroup was labeled by different continents and haplogroups with different colors (Supplementary Figure S8). Within the macrohaplogroup N, seven Kirgiz individuals clustered together and separated from the individuals of other reference populations.

## Discussion

In this research, mtDNA control region sequences were detected from 91 unrelated Kirgiz individuals to analyzed the genetic background of Kirgiz group located in the Chinese Northwest region, and laid the foundation for the mtDNA control region sequences in forensic practice applications and enriched the genetic information database of the Chinese populations. As a result, the Kirgiz group displayed relatively high genetic polymorphisms with the parameters including the number of haplotypes (h: 81), haplotype and nucleotide diversities (Hd:0.997 and Pi: 0.00842), and the average number of nucleotide differences (k:9.017) as Zimmermann said (Zimmermann et al., 2019). The 91 unrelated individuals can be separated from each other based on the mtDNA control region sequences, which might indicate the effectiveness of the 1122bp control region sequences for forensic maternal lineage analyses according to the recommendation of previous study (Cardoso et al., 2013). The neutrality test, mismatch distribution, median network analyses, and BSP inference are the most commonly used methods to evaluate the population historical dynamics and the population expansion time in the biogeographic analysis. The results of Tajima’s D test (-1.950, *p*-value < 0.05) and Fu’s FS test (-25.247, *p*-value < 0.05) indicated that the Kirgiz group experienced population expansion during its history as the previous study recommended (Ramos-Onsins and Rozas, 2002). The small and insignificant r values (raggedness index, 0.00689 of sudden expansion model and 0.00689 of spatial expansion model) and SSD indexes (0.00182 of sudden expansion model and 0.00216 of spatial expansion model) of the appropriateness test of the mismatch distribution also supported the hypothesis of population expansion of the Kirgiz group (Slatkin and Hudson, 1991). The expansion of the Kirgiz group occurred at about *53.41 kya* based on the curve of the BSP when the ancestries of early modern Kirgiz group began to expand. At that time, the individual fossils representing modern Asians and modern Europeans were discovered (Liu et al., 2021). Additionally, it was difficult to achieve smaller scale estimation of the expansion time to track smaller population expansion event because of the limited genetic information.

The population pairwise *F*_*ST*_ values were calculated based on haplogroup frequencies. And the *F*_*ST*_ heatmap showed the genetic differentiations between Kirgiz group and the reference populations. In the NJ phylogenetic tree, most of populations from the same continent clustered together except the immigrant populations like African America and Caucasians from North America. In addition, the Kirgiz group had the three smallest *F*_*ST*_ values with CHB, EPK and CHS populations from China, which indicated relatively close genetic affinities between Kirgiz and these three populations according to the criteria of genetic differentiations of Wright (Stoneking and Delfin, 2010). The largest three *F*_*ST*_ values were observed between Kirgiz group and PEL, VAL and CLM populations, which might indicate high differentiations and relatively remote genetic distances between Kirgiz group and these populations. What’s more, the previous study indicated that the Kirgiz was a mixed Eurasian group (Fahu Chen et al., 2019), and the present results of the coefficient analysis of genetic differentiations also showed that the Kirgiz was close to the East Asia populations. Subsequent median network analyses based on the mtDNA control region sequences also showed the similar results mentioned above. No matter considering the homologous haplogroup, directly connection or apart from one median vector, the East Aisa individuals had the most individuals who had close genetic affinities with the Kirgiz individuals. Several previously reported studies based on autosomal InDel markers and Y-STRs and SNPs (Guo et al., 2020; Song et al., 2021; Zhang et al., 2021) also supported that the Kirgiz had the close genetic relationships with the East Asia populations.

In summary, we provided 91 high quality mtDNA control region sequences of the Kirgiz ethnic group located in Northwest China. Fifty-two local mutations and three unexpected global mutations (199M, 451G, 502del) were identified among these mtDNA control region sequences of Kirgiz individuals. In addition, we evaluated the mtDNA genetic diversities in the Kirgiz group and gained insight into its genetic relationships with 48 reference populations all over the world. This study on the genetic diversities of mtDNA control region sequences in Kirgiz ethnic group might promote the understanding the genetic background of Kirgiz ethnic group and lay the foundation for the researches of maternal pedigrees, and promote the case investigations involving matrilineal families.

## Data Availability

The original contributions presented in the study are included in the article/Supplementary Material, further inquiries can be directed to the corresponding author.
