# Supplementary figures and images for: Genetic Background of Kirgiz Ethnic Group From Northwest China Revealed by Mitochondrial DNA Control Region Sequences on Massively Parallel Sequencing
Source: Front Genet. 2022 Feb 23;13:729514. doi: 10.3389/fgene.2022.729514 (PMC8906502; doi:10.3389/fgene.2022.729514)

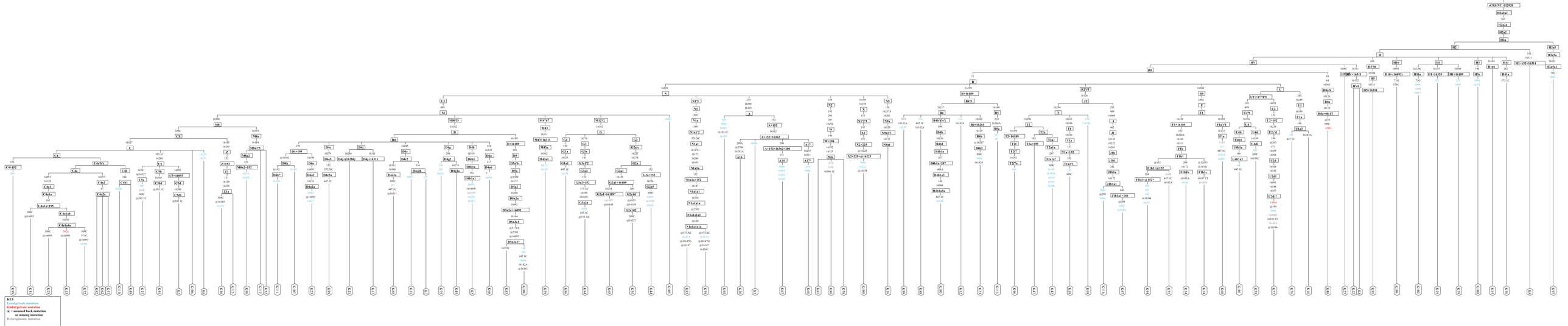

Supplement: Supplementary file 2 [file Image1.tiff]

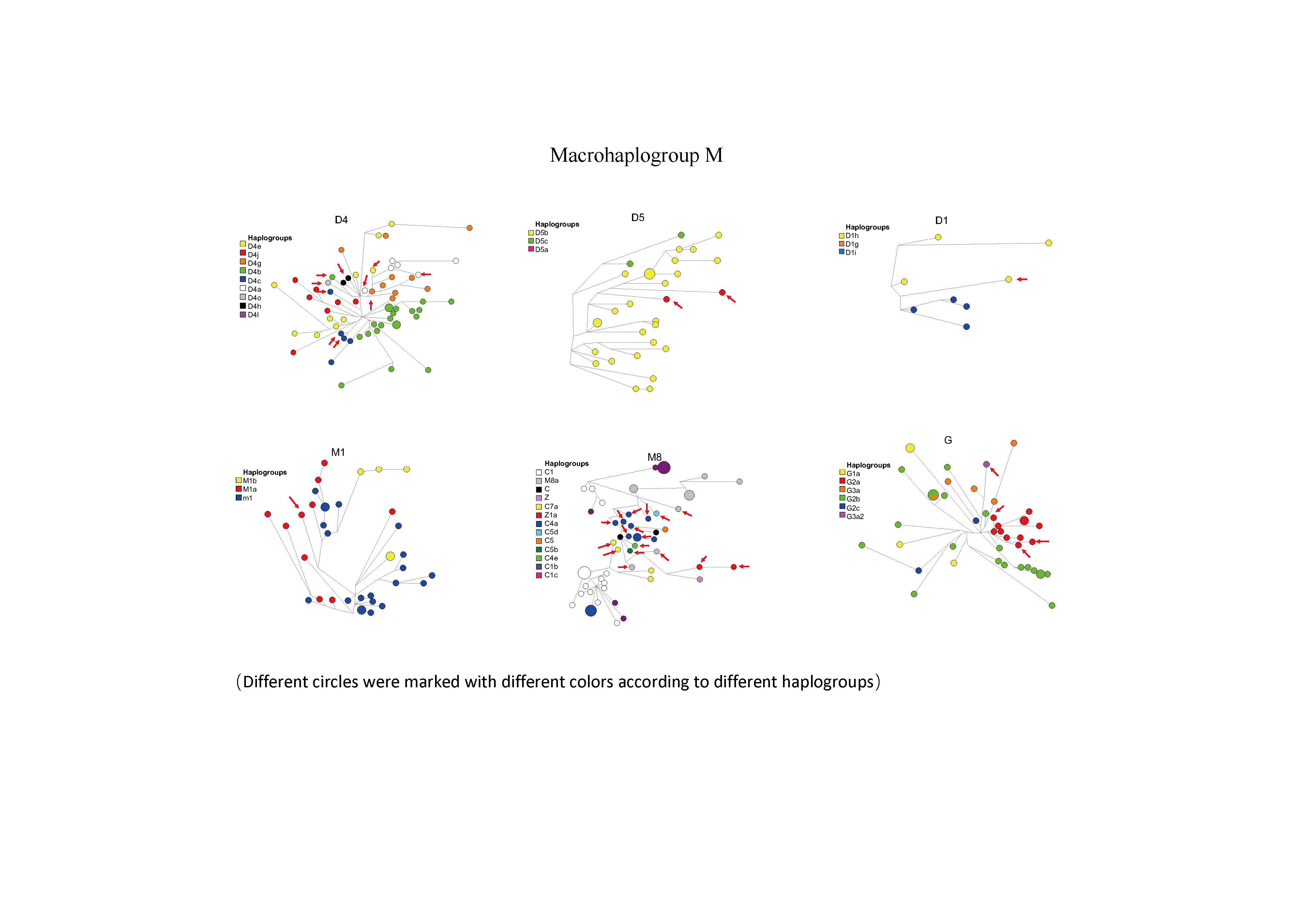

Supplement: Supplementary file 3 [file Image5.tiff]

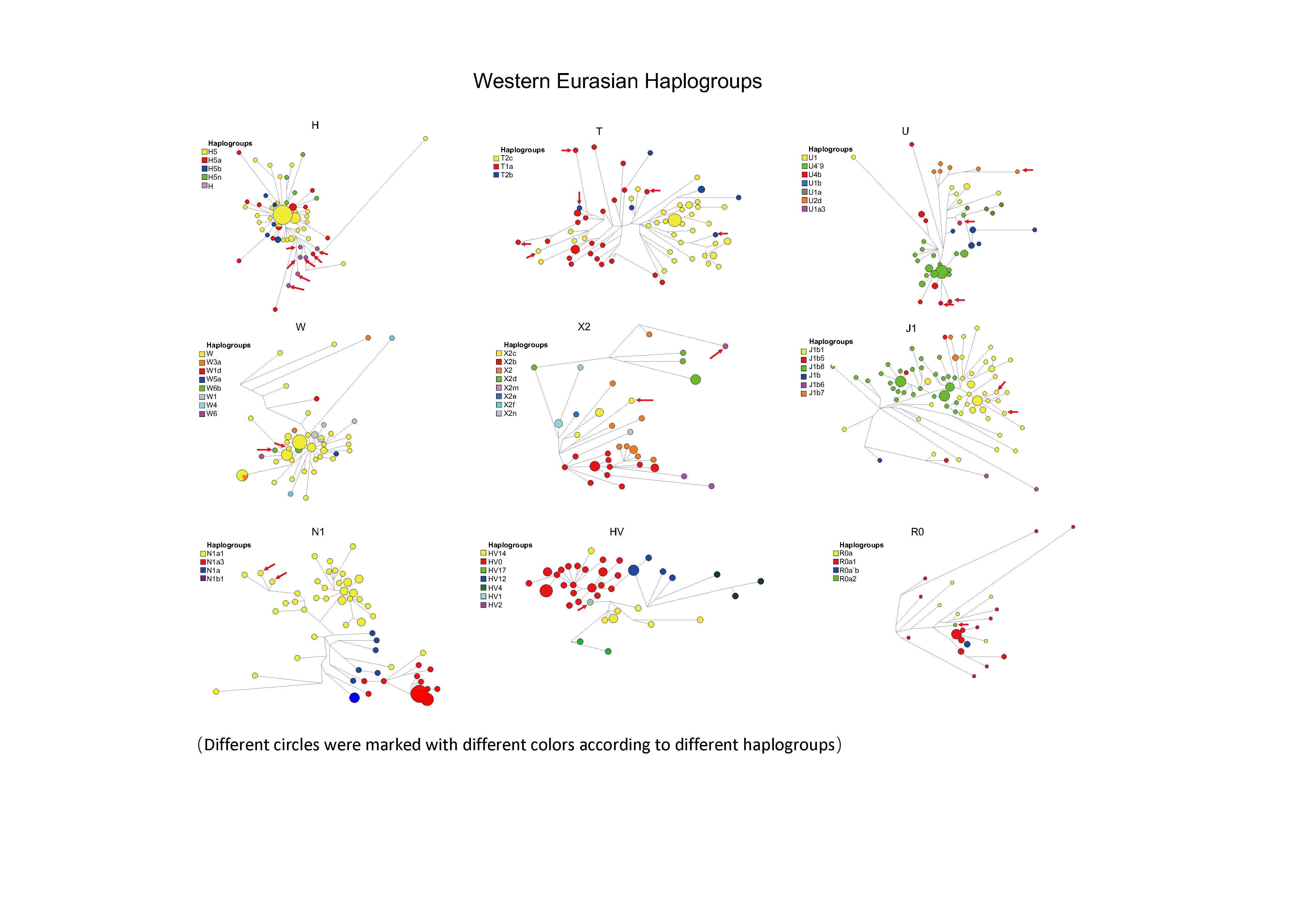

Supplement: Supplementary file 4 [file Image6.tiff]

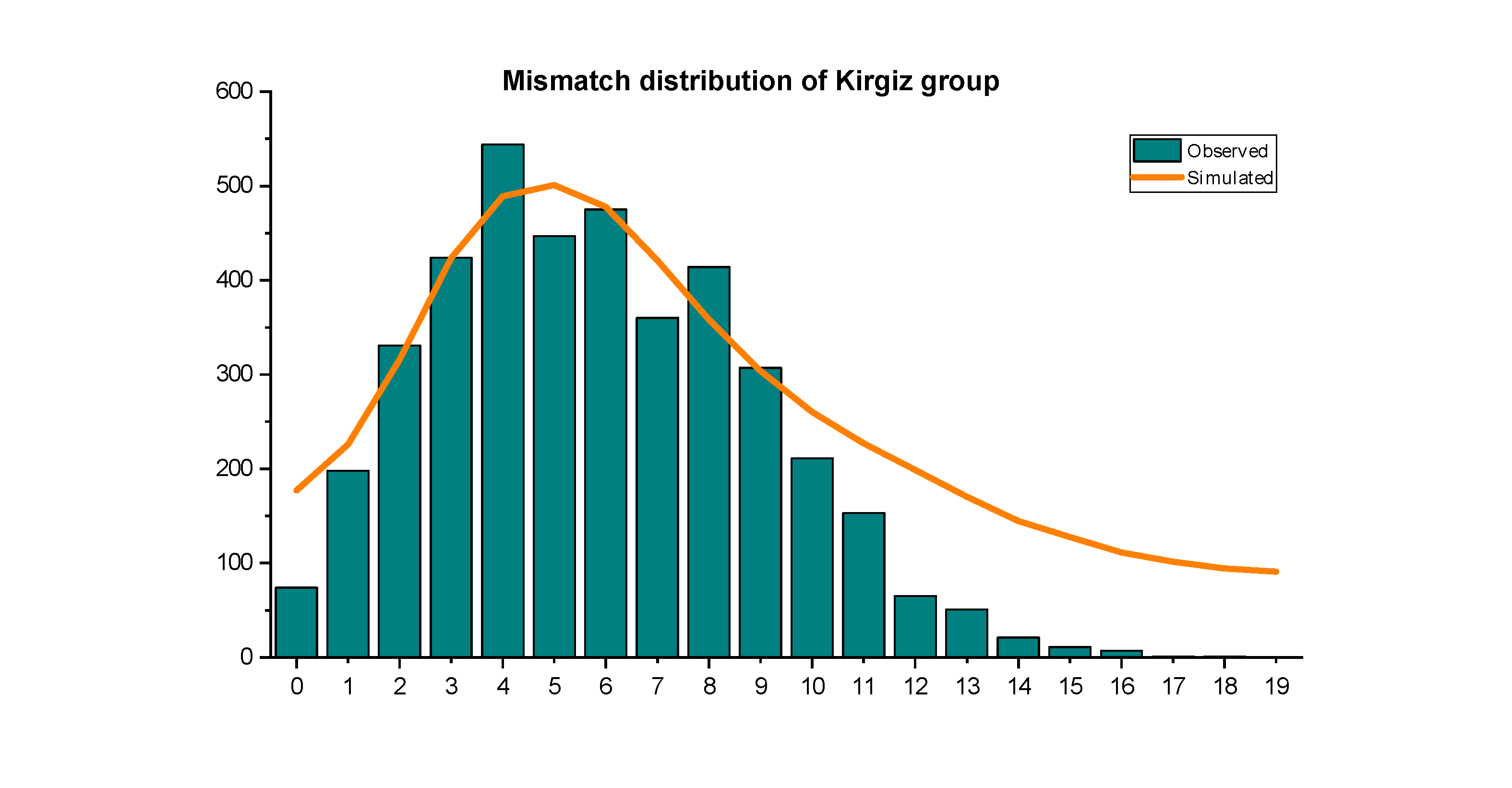

Supplement: Supplementary file 5 [file Image2.tiff]

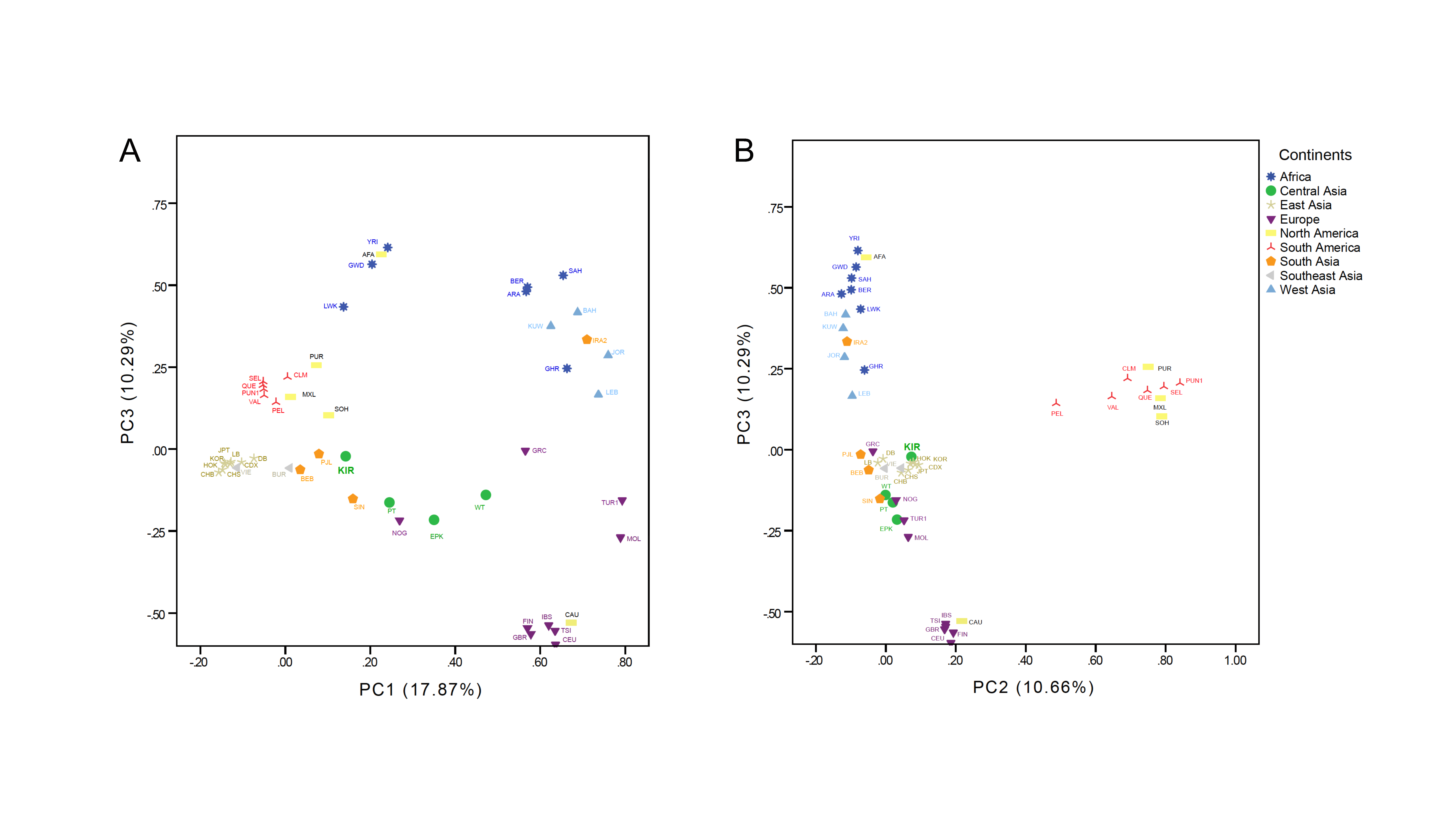

Supplement: Supplementary file 6 [file Image4.tiff]

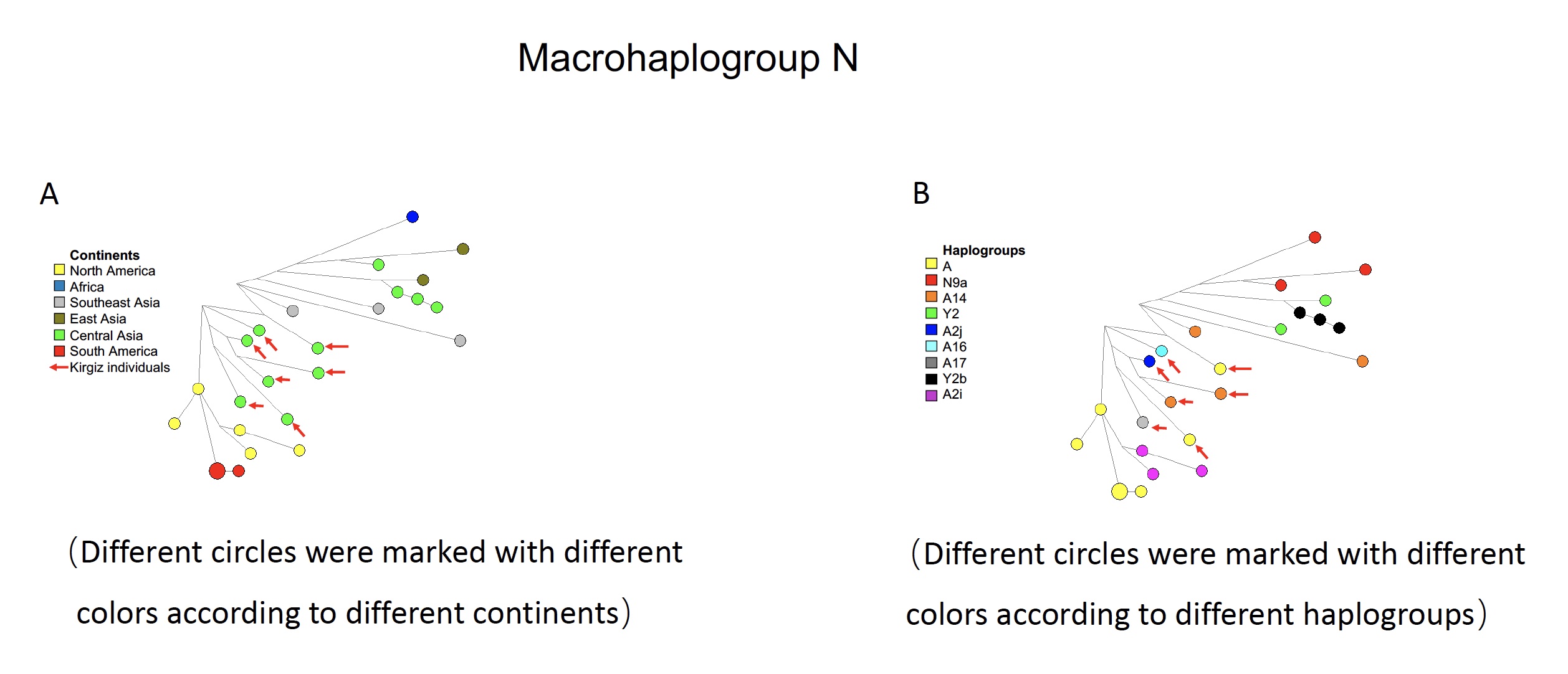

Supplement: Supplementary file 7 [file Image8.jpg]

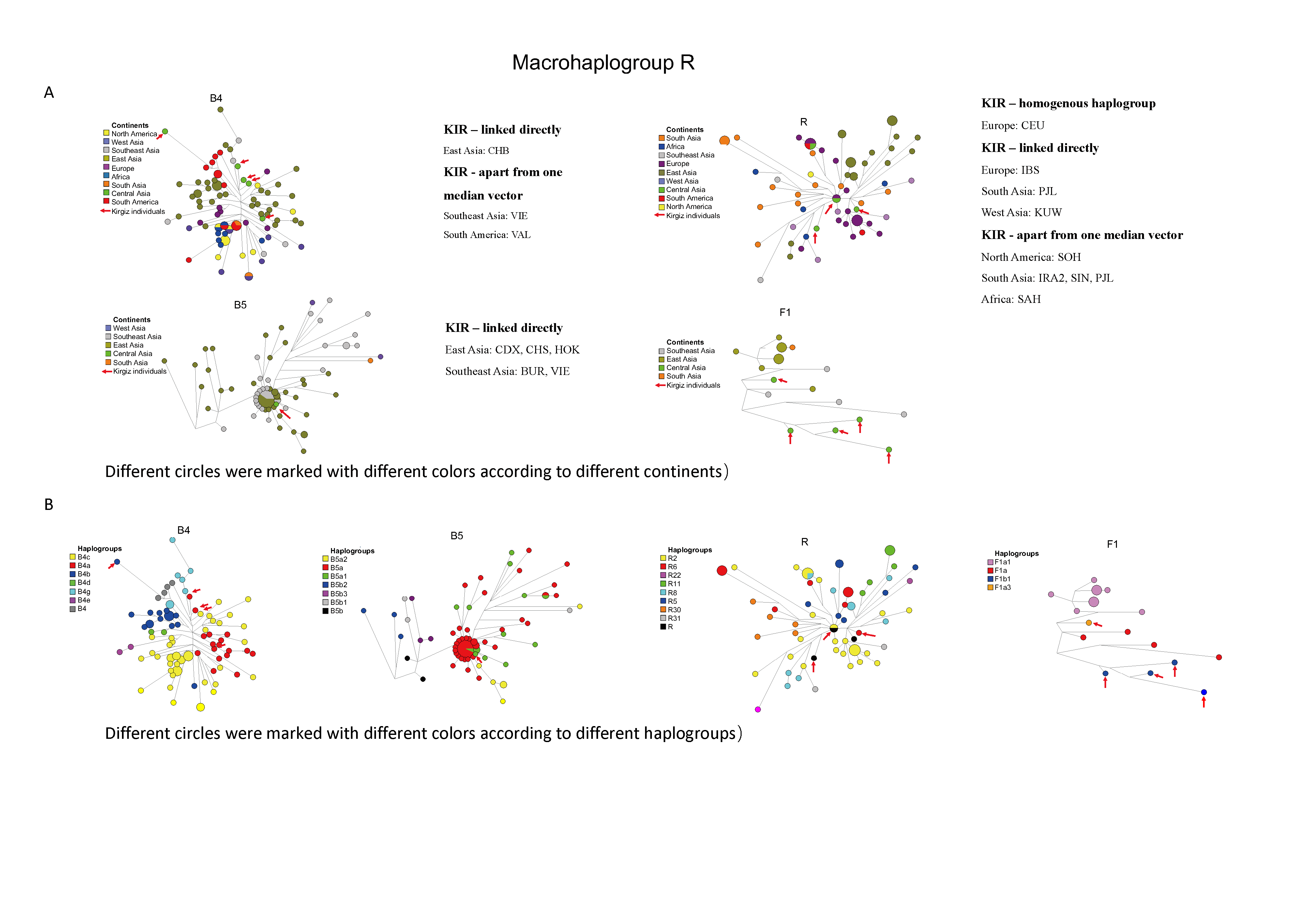

Supplement: Supplementary file 8 [file Image7.tiff]
